# Targeting the Full Length of the Motor End Plate Regions in the Mouse Forelimb Increases the Uptake of Fluoro-Gold into Corresponding Spinal Cord Motor Neurons

**DOI:** 10.3389/fneur.2013.00058

**Published:** 2013-05-20

**Authors:** Andrew Paul Tosolini, Rahul Mohan, Renée Morris

**Affiliations:** ^1^Translational Neuroscience Facility, School of Medical Sciences, University of New South WalesSydney, NSW, Australia

**Keywords:** motor end plates, motor neurons, Fluoro-Gold, mouse forelimb, motor neuron columns, retrograde tracing

## Abstract

Lower motor neuron dysfunction is one of the most debilitating motor conditions. In this regard, transgenic mouse models of various lower motor neuron dysfunctions provide insight into the mechanisms underlying these pathologies and can also aid the development of new therapies. Viral-mediated gene therapy can take advantage of the muscle-motor neuron topographical relationship to shuttle therapeutic genes into specific populations of motor neurons in these mouse models. In this context, motor end plates (MEPs) are highly specialized regions on the skeletal musculature that offer direct access to the pre-synaptic nerve terminals, henceforth to the spinal cord motor neurons. The aim of this study was two-folded. First, it was to characterize the exact position of the MEP regions for several muscles of the mouse forelimb using acetylcholinesterase histochemistry. This MEP-muscle map was then used to guide a series of intramuscular injections of Fluoro-Gold (FG) in order to characterize the distribution of the innervating motor neurons. This analysis revealed that the MEPs are typically organized in an orthogonal fashion across the muscle fibers and extends throughout the full width of each muscle. Furthermore, targeting the full length of the MEP regions gave rise labeled motor neurons that are organized into columns spanning through more spinal cord segments than previously reported. The present analysis suggests that targeting the full width of the muscles’ MEP regions with FG increases the somatic availability of the tracer. This process ensures a greater uptake of the tracer by the pre-synaptic nerve terminals, hence maximizing the labeling in spinal cord motor neurons. This investigation should have positive implications for future studies involving the somatic delivery of therapeutic genes into motor neurons for the treatment of various motor dysfunctions.

## Introduction

Knowledge regarding the organization of the lower motor neuron system has significantly developed through the work of Sherrington (1892), Romanes (1941, 1946, 1951), and Rexed (1954). Collectively, their work has established that motor neurons in the ventral horn of the spinal cord that innervate skeletal muscles are arranged into longitudinal columns. More recently, retrograde tracers, either applied to the peripheral nerve stump or injected intramuscularly, have been instrumental in defining the connectivity between individual skeletal muscles and the innervating motor neuron columns in various mammalian species (Kristensson and Olsson, 1971a,b; McHanwell and Biscoe, 1981; Jenny and Inukai, 1983; Nicolopoulos-Stournaras and Iles, 1983; Brichta et al., 1987; Callister et al., 1987; Hörner and Kümmel, 1993; Novikova et al., 1997; Vanderhorst and Holstege, 1997; McKenna et al., 2000; Choi et al., 2002; Tosolini and Morris, 2012; Bácskai et al., 2013a,b). Together, these studies further characterize the organization of motor neuron columns throughout the spinal cord.

Dysfunctions or diseases of the lower motor neurons are amongst the most debilitating motor disorders. In this regard, the emergence of numerous transgenic mouse models of lower motor neuron conditions provide insight into the mechanisms underlying these pathologies (Gurney et al., 1994; Wong et al., 1995, 2002; Hsieh-Li et al., 2000; Kaspar et al., 2003; Ishiyama et al., 2004; Turner et al., 2009; Wegorzewska et al., 2009; Kimura et al., 2010; Towne et al., 2010; Xu et al., 2010; Guo et al., 2011; Riboldi et al., 2011; Pratt et al., 2013). For example, the Cu/Zn superoxide dismutase type-1 (SOD-1) mouse model was developed in order to further understand the etiology and pathogenesis of a subtype of amyotrophic lateral sclerosis (Gurney et al., 1994; Wong et al., 1995; Raoul et al., 2005; Zhong et al., 2009; Towne et al., 2010; Riboldi et al., 2011). This is also the case for the survival motor neuron 1 (SMN) knockout mouse model of spinal muscular atrophy (SMA) (Hsieh-Li et al., 2000). With these mouse models, viral-mediated gene therapy can take advantage of the muscle-motor neuron topographical relationship to retrogradely shuttle therapeutic genes into specific populations of motor neurons (for recent reviews, see Bo et al., 2011; Wang et al., 2011; Federici and Boulis, 2012; Franz et al., 2012; Lentz et al., 2012). This approach has been explored using intramuscular bolus injections (Baumgartner and Shine, 1997, 1998; Kaspar et al., 2003; Nakajima et al., 2008, 2010; Uchida et al., 2012; Benkhelifa-Ziyyat et al., 2013). With this gene delivery method, however, the levels of transgene expression in motor neurons and, therefore, the outcomes of the therapy often remain suboptimal.

Motor end plates (MEPs) are highly specialized regions on the skeletal musculature that offer direct access to the pre-synaptic nerve terminals, henceforth to the spinal cord motor neurons. We have recently described the location and span of the MEP regions for several muscles of the rat forelimb (Tosolini and Morris, 2012). Targeting the entire MEP region with retrograde tracers has revealed that the motor neuron columns supplying the rat forelimb span more cervical segments and exhibit greater overlap with neighbor columns than previously reported (Tosolini and Morris, 2012). The aim of the present investigation was to extend this knowledge to the mouse, the species of choice for gene targeting in animal models of various motor conditions (Hsieh-Li et al., 2000; Kaspar et al., 2003; Ishiyama et al., 2004; Turner et al., 2009; Wegorzewska et al., 2009; Kimura et al., 2010; Towne et al., 2010; Xu et al., 2010; Guo et al., 2011; Riboldi et al., 2011; Pratt et al., 2013).

## Materials and Methods

### Animals

All experimental procedures complied with the Animal Care and Ethics Committee of the University of New South Wales and were performed in accordance with the National Health and Medical Research Council of Australia regulations for animal experimentation. A total of 38 adult male C57BL/6 mice (ARC, Western Australia) weighing between 20 and 30 g at the time of surgery were used in this study. The mice were housed in groups of five in an animal holding room under 12-h light–dark cycle. Water and chow were available *ad libitum* throughout the course of the experiment.

### Acetylcholinesterase histochemistry

Acetylcholinesterase histochemistry (AChE) was performed on mice carcasses as per Tosolini and Morris (2012). Six lightly perfused mice were obtained through tissue sharing. The skin was removed from the carcasses and the entire bodies were immersed for 4 h at 4°C in a solution containing 200 ml of phosphate buffer (PB), 290 mg acetylthiocholine iodide, 600 mg glycine, and 420 mg copper sulfate (all reagents from Sigma-Aldrich, St. Louis, MO, USA). The carcasses were subsequently washed for 2 min in distilled water and developed by rapid immersion (i.e., 5–10 s) in a 10% ammonium sulfide solution.

### Surgery

Anesthesia was induced with isoflurane (Provet, Sydney, NSW, Australia; 1–2% in O_2_). The fur covering the targeted areas was shaved and cleaned with 70% ethanol. For each muscle under investigation, a small incision was made directly in the skin to expose the muscle of interest. Fluoro-Gold (FG) (Fluorochrome, Denver, CO, USA) injections were manually performed through graded glass micropipettes (DKSH, Zurich, Switzerland) along the entire MEP region. Great care was taken to preserve the fasciae covering both the targeted muscles and those in the surrounding. Special care was also taken to ensure that the blood vessels surrounding the muscles were left intact. After the injections, the muscles were wiped with gauze to remove any tracer that may have inadvertently seeped from the injected muscle. A total of 47 series of intramuscular injections along the full extent of the MEP region were performed into the following muscles: acromiotrapezius (*n* = 6), acromiodeltiodeus (*n* = 6), spinodeltoideus (*n* = 5), biceps brachii (*n* = 6), triceps brachii (*n* = 6), extensor carpi ulnaris (*n* = 4), extensor carpi radialis (*n* = 6), flexor carpi ulnaris (*n* = 4), and flexor digitorum profundus (*n* = 4). For these injections, the volume of FG varied between 2 and 6 μl depending on the size of the muscle (i.e., triceps brachii received 6 μl whereas extensor carpi ulnaris received 2 μl). Triceps brachii was also targeted with either a 3-μl bolus injection of FG into the thickest part of the muscle (*n* = 6) or with 3 μl injections restricted to the anterior or the posterior portion of its MEP (*n* = 4). In additional animals, 3 μl of FG was applied directly onto the intact fasciae covering triceps brachii (*n* = 4). The skin was subsequently closed with surgical clips (Texas Scientific Instruments LLC, Boerne, TX, USA).

### Histological processing and dissection

After the intramuscular injections of FG, the mice were kept for 7 days to allow for optimal retrograde transport of the neuronal tracer. After this period of time, the mice received a lethal dose of Lethabarb (Virbac, Sydney, NSW, Australia) and were intracardially perfused with 0.1 M PB followed by 4% paraformaldehyde in 0.1 M PB. Dissections of the spinal cord were made from the dorsal aspect whereby the paravertebral muscles were reflected/removed and the cervical vertebral column was exposed. The bony spinous process of C2 was identified and then removed, exposing the C2 dorsal roots, which were then colored with a permanent marker. Vertebrae C3–T1 were subsequently removed one by one and the dorsal roots were colored in alternating colors (i.e., C2, C4, C6, and C8 were colored with a green marker and C3, C5, C7, T1 were colored with a blue marker). After this process, the cervical spinal cord was cut transversely into two-segment blocks (i.e., C2–C3, C4–C5, C6–C7, and C8–T1 blocks). For each block, a fiducial mark was made *in situ* in the white matter, half way between two adjacent roots to indicate the boundary between the segments. The blocks were then removed from the body, post-fixed overnight in a solution containing 4% paraformaldehyde in 0.1 M PB and then cryoprotected in a 30% sucrose solution (Sigma-Aldrich, St. Louis, MO, USA) in distilled water for 2 days at 4°C. Each block of spinal cord tissue was cut longitudinally in 50 μm-thick sections and mounted onto microscope slides. The slides were air-dried and then coverslipped with an anti-fade medium containing DAPI (Invitrogen, Carlsbad, CA, USA).

### Data analysis and presentation

After the AChE procedure, the bodies were photographed, and Adobe Photoshop CS6 was used to transpose the average locations of the MEPs onto a diagrammatic representation of the mouse forelimb adapted from Komárek (2004) and DeLaurier et al. (2008).

The spinal cord tissue sections were photographed and analyzed under epifluorescence to detect FG-labeled motor neurons. Motor neurons were considered positively labeled when FG granulations were present within both the soma and at least one axon/dendrite (Vanderhorst and Holstege, 1997; Tosolini and Morris, 2012; Bácskai et al., 2013a,b). Adjacent tissue was also scrutinized to eliminate double counting of motor neurons. For each tissue section, FG labeled motor neurons were plotted as single black dots on a separate layer of a diagrammatic representation of the spinal cord using Adobe Photoshop CS6. Root exit points, the position of the central canal and the fiducial marks created during dissection were used as spatial references. The Adobe Photoshop layers were subsequently stacked together to create a single two-dimensional representation of the position of the motor neurons innervating each forelimb muscle. For each muscle, individual data plots were then presented side by side on a schematic diagram of a spinal cord (Figure 3). The data plots derived from intramuscular injections performed on the left forelimb were transposed onto the right spinal cord to maintain consistency with the representation. For all muscles, data plots were then combined to form a representative motor neuron column and were represented concurrently in rostro-caudal, dorso-ventral, and medio-lateral axes in the same figure (Figure 5).

## Results

### Motor end plate delineation

**Figure 1:**
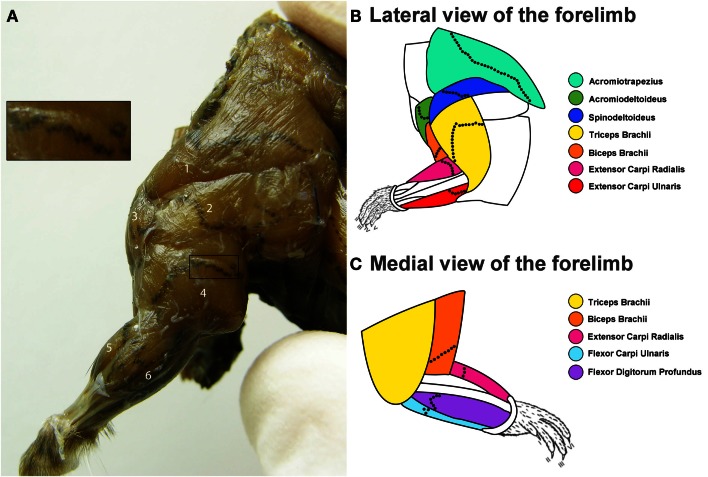
**Distribution of the motor end plate (MEP) regions for the mouse forelimb**. **(A)** Lateral view of the mouse forelimb after an aceytlcholinesterase (AChE) histochemical reaction to reveal the location of the MEPs. In this figure, the MEPs appear as black speckles traversing the brown muscle fibers. From this lateral view, the following muscles can be seen: (1) acromiotrapezius, (2) spinodeltoideus, (3) acromiodeltoideus, (4) triceps brachii, (5) extensor carpi radialis, and (6) extensor carpi ulnari. The insert shows a close up view of the MEPs from a portion of the triceps brachii muscle. **(B,C)** Composite diagrams representing the location of the MEPs from the lateral **(B)** and medial **(C)** view of the forelimb. The color-coded forelimb muscles targeted are: acromiotrapezius (turquoise), acromiodeltoideus (green), spinodeltoideus (dark blue), biceps brachii (orange), triceps brachii (yellow), extensor carpi ulnaris (red), extensor carpi radialis (magenta), flexor carpi ulnaris (light blue), and flexor digitorum profundus (purple). The black dotted lines on each muscle are representative locations of the MEP region.

Overall, for each muscle the location of the MEP region was similar between animals. The location and span of the MEPs for each muscle investigated is shown in Figure 1. Figure 1A is a photograph showing the lateral view of a mouse forelimb after an AChE reaction. The MEPs can be seen as black speckles traversing the muscle fibers. On this photograph, the boundaries of each muscle as well as the direction of their muscle fibers can also be observed, allowing for *in situ* muscle orientation. Figures 1B,C are schematic representations of the lateral and medial forelimb on which the location of the MEPs were transposed. The MEPs are typically organized in an orthogonal fashion across the muscle fibers. The MEP regions can be seen as extending across the full width of the muscles, passing through, but not limited to the region commonly referred to as the muscle “belly” (Figures 1A–C). It is worthwhile to note that some muscles do not have a “belly” region but still have a clearly observable MEP region. This is the case for acromiotrapezius, spinodeltoideus, and acromiodeltoideus. As each muscle has its own shape, so too does its MEP region. In most instances, the MEP regions are sinusoidal-like or V-shaped, however they never appeared to form a straight line. For muscles with multiple heads such as triceps and biceps brachii, the MEP region is located in the common part of the muscle, not in the heads. The thin and narrow muscles, such as those acting on the wrist joint (extensor carpi radialis, extensor carpi ulnaris, flexor digitorum profundus, and flexor carpi ulnaris) have their MEP region located closer to the elbow joint rather than in the middle of the muscle (Figures 1A–C).

### Fluoro-Gold labeled motor neurons

**Figure 2:**
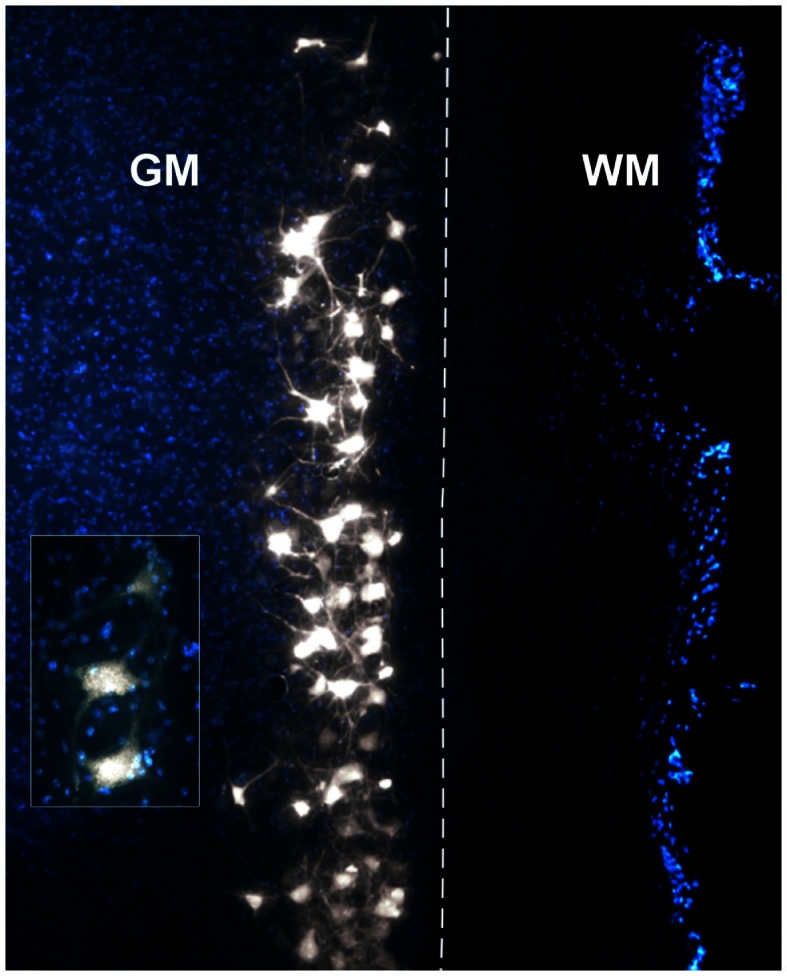
**Photomicrograph of a DAPI-stained longitudinal section through a right cervical spinal cord demonstrating a typical column of Fluoro-Gold (FG)-labeled motor neurons**. The insert displays a higher magnification of motor neurons with clear FG granulations in the somas and axons/dendrites. GM, gray matter; WM, white matter. The dashed line represents the border between GM and WM.

The intramuscular injections of FG gave rise to intense labeling of motor neurons in proximity to the border between the gray and white matter. Figure 2 is a photomicrograph of a right cervical cord to illustrate a typical column of labeled motor neurons. On this figure, FG granulations are present within multiple motor neuron somas and their processes.

### Distribution of motor neuron columns supplying individually targeted muscles

A total of nine forelimb muscles were targeted with intramuscular injections of FG: three muscles acting on the shoulder joint (acromiotrapezius, acromiodeltoideus, and spinodeltoideus), two muscles acting on the elbow joint (biceps brachii and triceps brachii), and four muscles acting on the wrist joint (extensor carpi radialis, extensor carpi ulnaris, flexor carpi ulnaris, and flexor digitorum profundus).

#### Acromiotrapezius

**Figure 3:**
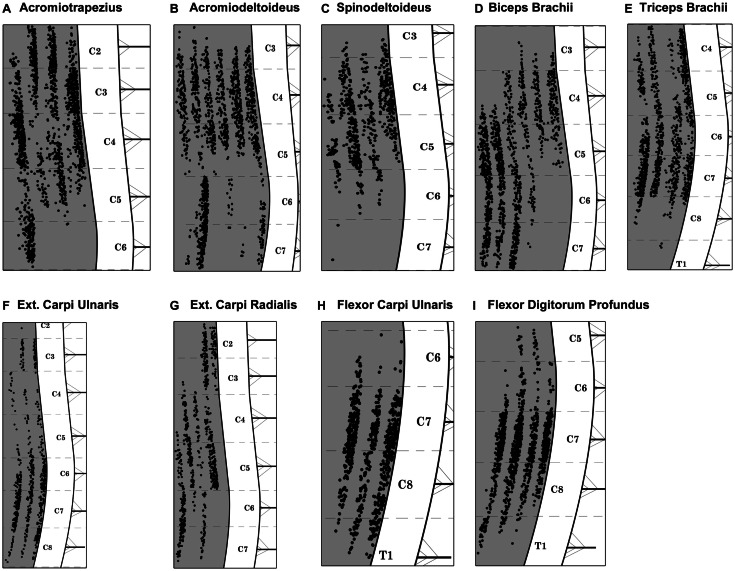
**Composite diagram illustrating the distribution of labeled motor neurons from each targeted muscle**. Each black dot represents one labeled motor neuron and each columnar-shaped data set represents the FG-labeling observed after intramuscular injections in one muscle. **(A)** Acromiotrapezius, **(B)** acromiodeltoideus, **(C)** spinodeltoideus, **(D)** biceps brachii, **(E)** triceps brachii, **(F)** extensor carpi ulnaris, **(G)** extensor carpi radialis, **(H)** flexor carpi ulnaris, and **(I)** flexor digitorum profundus. Spinal cord levels are indicated in the white matter on the right hand side of each diagram. Each cervical/thoracic spinal cord segment is demarcated by dashed lines. These lines correspond to the halfway point between two nerve roots.

Acromiotrapezius, one of the muscles forming the trapezius muscle group (Komárek, 2004; DeLaurier et al., 2008), can be seen on the lateral aspect of the mouse forelimb (Figures 1A,B). Acromiotrapezius is a thin but large muscle connecting the spinous processes of vertebrae to the acromion process of the scapula. It is involved in retraction, elevation, and depression of the scapula. Six series of injections were performed along the MEP region of acromiotrapezius, four of which gave rise to intense labeling in the ventral horn of the cervical spinal cord. Data from these successful series injections (*n* = 4) were included in the present analysis. Such injections resulted in labeled motor neurons forming a column spanning segments C2–C6 of the spinal cord (Figure 3A).

#### Acromiodeltoideus

Acromiodeltoideus, one of the two muscles comprising the deltoid muscle group (Komárek, 2004; DeLaurier et al., 2008), is located on the lateral aspect of the mouse forelimb (Figures 1A,B). Acromiodeltoideus also acts on the glenohumeral joint. It is a small triangular muscle located at the anterior point of the shoulder at the junction of spinodeltoideus and biceps brachii (Figures 1A,B). Six series of injections of FG were performed in acromiodeltoideus and all six gave rise to intense labeling spanning segments C3–C7 of the cervical spinal cord (Figure 3B). Data from these successful series of injections (*n* = 6) were included in the present analysis.

#### Spinodeltoideus

Together with acromiodeltoideus, spinodeltoideus belongs to the deltoid muscle group (Komárek, 2004; DeLaurier et al., 2008). Spinodeltoideus is a trapezoidal-shaped muscle present on the lateral surface of the mouse forelimb (Figures 1A,B), immediately adjacent to acromiotrapezius. As is the case with acromiotrapezius and acromiodeltoideus, spinodeltoideus acts on the glenohumeral joint. Five series of intramuscular injections were performed on spinodeltoideus. Of these five series of injections, four series gave rise to bright labeling of motor neurons spanning segments C3–C6 of the cervical spinal cord (Figure 3C). Data from these four successful injections (*n* = 4) were included in the present analysis. It is worthwhile to mention that, in one case, two labeled motor neurons were present in C7. These two motor neurons were not taken into account in further analysis.

#### Biceps brachii

Biceps brachii is located on the ventral aspect of the upper forelimb (Figures 1A–C) (Komárek, 2004; DeLaurier et al., 2008). The two proximal heads of biceps brachii unite to form one distal muscle mass that flexes the elbow joint. A total of six series of intramuscular injections of FG were performed into biceps brachii and all of the series gave rise to intense labeling of motor neurons between segments C3 and C7 of the spinal cord (Figure 3D). Data from these six successful series of injections (*n* = 6) were included in the present analysis.

#### Triceps brachii

Triceps brachii is located on the dorsal aspect of the upper forelimb (Figures 1A–C) (Komárek, 2004; DeLaurier et al., 2008). Similarly to biceps brachii, the three proximal heads of triceps brachii join to form one belly that extends the elbow joint. A total of six series of intramuscular injections of FG were performed into triceps brachii, four series of which resulted in intense labeling of a motor neuron column that spans segments C4–T1 of the spinal cord (Figure 3E). Data from these four series of injections (*n* = 4) were included in the present analysis. In one series case, however, three labeled motor neurons were present in rostral T1. These three motor neurons were not taken into account in further analysis.

#### Extensor carpi ulnaris

Extensor carpi ulnaris is one of the two muscles targeted in the present investigation that extends the wrist joint (Komárek, 2004; DeLaurier et al., 2008). Extensor carpi ulnaris is a thin and shallow muscle located on the dorsal aspect of the distal forelimb (Figures 1A,B). Overall, there were four series of intramuscular injections performed in extensor carpi ulnaris, with three series giving rise to intense motor neuron labeling located between segments C2 and C8 of the cervical spinal cord (Figure 3F). Data from these three successful series of injections (*n* = 3) were included in the present analysis. In one case, however, one labeled motor neuron was present in caudal C2. This motor neuron was not taken into account in further analysis.

#### Extensor carpi radialis

Together with extensor carpi ulnaris, extensor carpi radialis, which is located on the dorsal part of the mouse distal forelimb, extends the wrist joint (Figures 1A–C) (Komárek, 2004; DeLaurier et al., 2008). Extensor carpi radialis is comprised of a smaller brevis and a larger longus compartments; however, both parts were targeted together. A total of six series of intramuscular injections of FG were performed into extensor carpi radialis. Of these six series, five series of injections gave rise to consistent labeling of motor neurons spanning cervical segments C2–C7 (Figure 3G). Data from these five injections (*n* = 5) were included in the present analysis.

#### Flexor carpi ulnaris

Flexor carpi ulnaris, a flexor muscle that acts on the wrist joint, is located on the ventral aspect of the distal part of the mouse forelimb (Figure 1C) (Komárek, 2004; DeLaurier et al., 2008). A total of four series of intramuscular injections of FG were performed in flexor carpi ulnaris. Of these injections, three gave rise to intense labeling of motor neurons forming a column between segments C6 and T1 of the spinal cord (Figure 3H). Data from these three injections (*n* = 3) were included in the present analysis.

#### Flexor digitorum profundus

As is the case for flexor carpi ulnaris, flexor digitorum profundus is a wrist flexor located on the ventral aspect of the mouse distal forelimb (Figure 1C) (Komárek, 2004; DeLaurier et al., 2008). A total of four series of intramuscular injections were performed into flexor digitorum profundus and all four series gave rise to intense motor neuron labeling between segments C5 and T1 of the spinal cord (see Figure 3I). Data from these four injections (*n* = 4) were included in the present analysis.

### Partial targeting of the motor end plate region in triceps brachii

**Figure 4:**
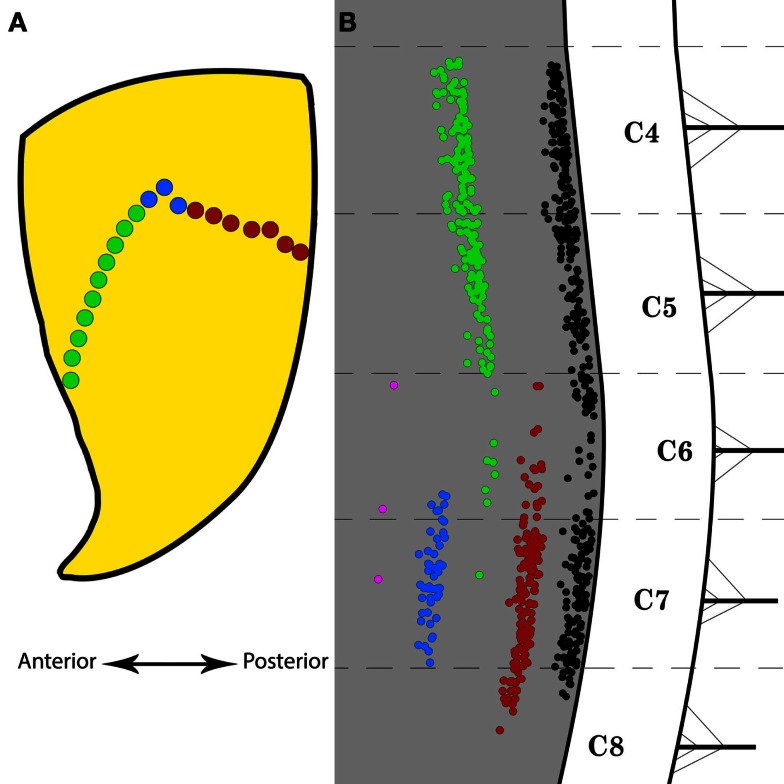
**Selective Fluoro-Gold (FG) targeting of the motor end plate region in triceps brachii and the resulting labeling in the spinal motor neurons**. **(A)** Schematic representation of the motor end plates (MEPs) selectively targeted on the triceps brachii muscle. The green and red dots represent the anterior and posterior halves of the entire MEP region, respectively. The blue dots indicate the location of a bolus injection of FG in the belly of the muscle. The double-headed arrow indicates the antero-posterior direction. **(B)** Distribution of labeled motor neurons resulting from selective MEP injections of FG as indicated in **(A)**. The black motor neuron column is taken from Figure 3E and represents the typical labeling observed after full-length MEP injections in triceps brachii. The red motor neuron column was obtained after FG injections along the posterior half of the MEP region. The green motor neuron column was obtained after FG injections along the anterior half of the MEP region. The blue motor neuron column was obtained after FG bolus injections in the belly of triceps brachii. The magenta “column” was obtained after application of FG onto the external surface of the fascia over triceps brachii. Each cervical/thoracic spinal cord segment is demarcated by dashed lines. These lines correspond to the halfway point between two nerve roots.

Figure 4A is a schematic representation of the portions of the MEP region of triceps brachii that were selectively targeted with FG. These regions are the anterior and posterior halves and the center of the MEP region. Figure 4B shows the distribution of labeled motor neurons resulting from these partial injections. Injections throughout the full length of the MEP region gave rise to columns of labeled motor neurons that extends from segment C4 through the rostral part of C8. In comparison, the partial targeting of the MEP region gave rise to correspondingly partial labeling of triceps brachii’s motor neuron column. More specifically, the targeting of the anterior portion of the MEP region resulted in the labeling of motor neurons forming a column mainly spanning segments C4–C5. Conversely, FG injections limited to the posterior part of the MEP region for triceps brachii gave rise to a column of positively labeled motor neurons, the bulk of which was confined within segments C7 and the rostralmost aspect of C8. Moreover, single injections of FG in the center of the MEP region produced labeling in fewer motor neurons mainly confined to segment C7. Furthermore, the application of FG onto the external surface of triceps brachii’s fascia resulted in a negligible number of labeled motor neurons.

### Overall organization of the motor neuron columns supplying the mouse forelimb

**Figure 5:**
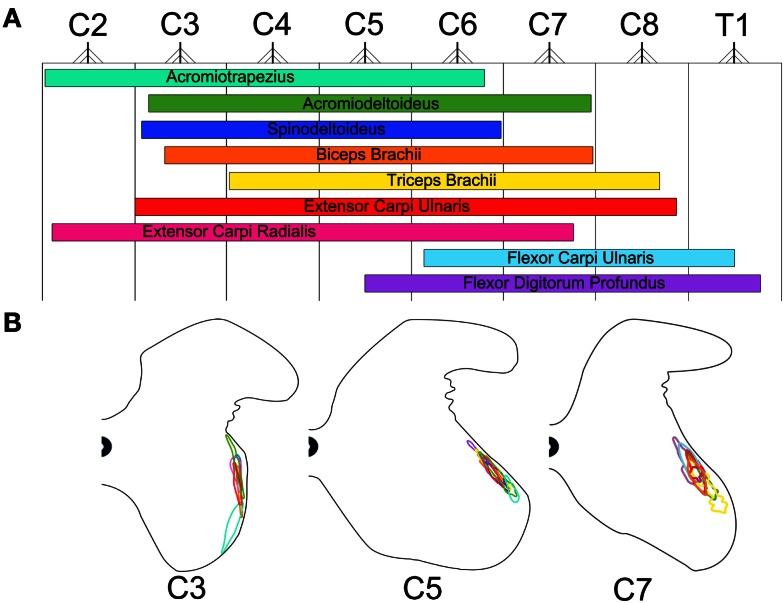
**Color-coded schematic map of the motor neuron columns innervating the Fluoro-Gold targeted forelimb muscles**. The color schemes remain consistent with that of Figures 1B,C. **(A)** Rostro-caudal map of the motor neuron columns innervating the targeted forelimb muscles. These columns were obtained by combining plots from Figure 3. The nerve root exit points represent the halfway point between spinal cord segments throughout C2–T1. **(B)** Dorso-ventral and medio-lateral map of the motor neuron columns innervating the targeted forelimb muscles for spinal cord segments C3, C5, and C7. The gray matter contours were adapted from Watson et al. (2009).

Figure 5 is a diagrammatic representation of the motor neuron columns for all muscles targeted in the present investigation. On the rostro-caudal axis, these columns of motor neurons encompass segments C2–T1 of the mouse spinal cord (Figure 5A). As shown in this figure, a great amount of overlap can be observed between these motor neuron columns. Motor neuron columns innervating the muscles acting on the glenohumoral joint (i.e., acromiotrapezius, acromiodeltoideus, and spinodeltoideus) extend from segments C2 to C7 whereas the motor neuron columns supplying the muscles acting on the elbow joint (i.e., biceps brachii and triceps brachii) span segments C3–C8. Moreover, the extensor (i.e., extensor carpi radialis, extensor carpi ulnaris) and flexor (i.e., flexor carpi ulnaris and flexor digitorum profundus) muscles acting on the wrist joint are innervated by motor neuron columns spanning segments C2–C8 and C5–T1, respectively. Overall, there is a topographical relationship, on the rostro-caudal axes, between the different muscles targeted in the present analysis and the motor neuron columns that innervate them. This relationship is such that the proximal-most muscles (e.g., acromiotrapezius) are innervated by motor neuron columns located in the rostral segments of the cervical spinal cord whereas the distal-most muscles (e.g., flexor carpi ulnaris) are supplied by columns located more caudally. Exceptions to this organizational scheme are the motor neuron columns for the two wrist extensors, namely extensor carpi radialis and extensor carpi ulnaris. Although these muscles are located in the distal part of the forelimb, they are supplied by motor neuron columns located in rostral segments of the spinal cord. Figure 5B shows the motor neuron columns in the transverse plane at spinal cord levels C3, C5, and C7. On this plane, the columns of motor neurons also exhibit a high level of overlap with each other. Overall, there is a dorso-ventral topographical relationship between the muscles targeted in the present investigation and the motor neurons that supply them. This relationship is such that the proximal (e.g., acromiotrapezius) and distal muscles (e.g., flexor carpi ulnaris) are innervated by motor neuron columns located ventrally and dorsally, respectively, within the ventral horn spinal cord.

## Discussion

We have recently targeted the entire MEP region of several muscles of the rat forelimb with retrograde tracers (Tosolini and Morris, 2012). The results of this analysis showed that the motor neuron columns that supply these muscles extend over more cervical spinal cord segments and display greater overlap with one another than formerly reported. The aim of the present investigation was to transfer this knowledge to the species of choice for the design of genetically engineered models of motor dysfunction, namely the mouse. The main outcomes of this study are first, the production of a detailed map of the motor end plate region for nine muscles of the mouse forelimb. This map was subsequently used as a guide to target the entire motor end plate region of these muscles with FG. The second main outcome is the characterization of the topographical organization that exists between the mouse forelimb muscles and the motor neuron columns that innervate them.

### Motor end plate analysis

To our knowledge, the present study is the first description of the MEP regions in the mouse forelimb. This analysis revealed that the MEP region for the different muscles targeted is located orthogonally to the direction of the muscle fibers. It is noteworthy that the MEP region is not consistently located within the fleshy part of a muscle (i.e., the muscle “belly”). Moreover, some muscles such as acromiotrapezius, spinodeltoideus, and acromiodeltoideus are flat and therefore do not have such a fleshy region. In these muscles as well as in triceps and biceps brachii, the MEP region traverses the entire width of the muscle (see Figure 1). The thin and narrow muscles, such as extensor carpi radialis, extensor carpi ulnaris, flexor digitorum profundus, and flexor carpi ulnaris have a fleshy part where the MEP regions are located. However, in these muscles, the so-called “belly” is not located in the center of the muscle but closer to the elbow joint. In most cases, the MEP region does not form a straight band. Rather, MEPs often exhibit a sinusoidal-like curve or are V-shaped. These findings are consistent with previous MEP characterization in the rat forelimb (Tosolini and Morris, 2012).

Fluoro-Gold was also injected along partial aspects of the MEP region in triceps brachii (see Figure 4A). The rostro-caudal extent of the labeling obtained from these partial MEP injections was then compared with that resulting from injections along the full length of the MEP region (see Figure 4B). Injections of FG restricted to the anterior half of the MEP region gave rise to a column of labeled motor neurons spanning only the rostral part of the columns of neurons obtained after complete MEP region injections. Conversely, FG injections restricted to the posterior half of the MEP region in triceps brachii produced a column of labeled motor neurons spanning only the caudal half of the column of motor neurons obtained after complete MEP region injections. Interestingly, combined labeling from the anterior and posterior MEP injections resulted in a column of similar span to that produced from the injections of the complete MEP region. These data suggest the existence of a MEP/motor neuron topographical relationship, although more data points need to be generated to confirm this *a priori* interesting finding. Should such organization be confirmed for triceps brachii, the MEP/motor neuron relationship will have to be established for the other muscles of the mouse forelimb. Injections selectively targeting the belly of triceps brachii were also performed (see Figure 4). As compared with the complete MEP data, these injections gave rise to shorter columns with substantially less FG-positive motor neurons. Together, these data can explain, at least partly, why targeting the entire MEP region with FG gave rise to labeled motor neurons spanning more spinal cord segments than recently observed (e.g., Bácskai et al., 2013a).

### Methodological considerations

In the present analysis, functionally diverse muscle groups that act on the three major joints of the forelimb were targeted with FG, namely the shoulder, elbow, and wrist. Superficial muscles of the mouse forelimb were selected for this tract-tracing experiment because these muscles are easily accessible. Indeed, the deeper muscles of the forelimb require a significant amount of dissection before they can be exposed and subjected to neuronal tracer injections, hence creating a risk for contamination of the tracers to surrounding muscles. It is our opinion that the superficial muscles of the forelimb have greater translational relevance than the deeper ones, as they are more likely to be the target in clinical trials involving somatic gene therapy.

In our hands, FG has proven to be a reliable and robust retrograde tracer that produces intense labeling of the neuronal somas and processes, therefore allowing for easy identification and direct count of motor neurons (see Figure 2). Importantly, unlike some other neuronal tracers, FG does not have the tendency to leak out of the cells (Schmued and Fallon, 1986). Yet, leakage has been recently reported after intramuscular injections of FG in the mouse forelimb (Bácskai et al., 2013a). It is important to note that muscle fasciae consist of tough layers of fibrous connective tissue that act as a natural barrier to tracer leakage (Haase and Hrycyshyn, 1986). In the present study, great care was taken to leave the muscle fasciae intact, aside from the penetrations of the ultra-thin micropipettes. It is also worth mentioning that the outermost aspect of the fasciae (i.e., the fasciae that can be readily visualized once the skin over the muscle of interest is cut open) was difficult to puncture even with the sharpest glass micropipettes. As the innermost fasciae (i.e., the fasciae that cover the innermost part of the muscles) would offer the same resistance against puncture, we are confident that the minute amount of tracer injected did not contaminate the deep muscles underlying those of interest. Additionally, the exposed region was routinely wiped off immediately after each injection in order to remove any tracer that may have seeped out. Special care was also taken to ensure that the blood vessels surrounding the muscles were not perforated. Taken together, these precautions ensured that insignificant spurious labeling was generated in the present investigation. However, leakage of the tracer to adjacent muscles cannot be entirely ruled out. To investigate the possibility that, despite these extensive precautions, some tracer might have been taken up by non-targeted surrounding muscles, FG was directly applied onto the external fascia covering triceps brachii (see Figure 4B). The result of such application of FG resulted in negligible labeling, therefore confirming that, at least in our hands, spurious labeling was not significant.

Overall, all injections of FG in the same muscles resulted in similar labeling for each animal. However, some variability was observed, in the extent, on the rostro-caudal axes, of the motor neuron column (see Figure 3). The same observation has been reported in previous tract-tracing investigations of the organization of motor neurons supplying the skeletal muscles (Hollyday, 1980; McHanwell and Biscoe, 1981; Nicolopoulos-Stournaras and Iles, 1983; Vanderhorst and Holstege, 1997; McKenna et al., 2000; Coonan et al., 2003; Tosolini and Morris, 2012; Bácskai et al., 2013a,b). This variability could be due to intraspecies differences with regard to the overall number of motor neurons innervating a muscle, a phenomenon that, in turn, influences the length and/or spatial distribution of the motor neuron columns. Interestingly, we also found slight differences in spatial distribution of motor neuron columns within the same animal, i.e., between homologous muscles in the left and right forelimbs. It is possible that this finding reflects forelimb use preference (i.e., handedness). Forelimb preference when reaching is well documented in the rat (Gharbawie et al., 2007; Alaverdashvili et al., 2008; Alaverdashvili and Whishaw, 2010; Morris et al., 2011); however, it is still a matter of debate in the mouse (Neveu et al., 1988; Takeda and Endo, 1993; Waters and Denenberg, 1994; Biddle and Eales, 1996; Bulman-Fleming et al., 1997). In the present study, forelimb use preference was not determined prior to the delivery of neuronal tracer. Thus, whether forelimb use preference affects the distribution of the motor neuron columns remains to be investigated. On the other hand, one cannot rule out that there could have been differences in the uptake of FG across different injections targeting the same muscle. Indeed, although great care was taken to minimize inter-injection variability, the uptake of FG could have been suboptimal in some cases. In these instances, the number of labeled motor neurons can actually be considered as an underestimation of the actual population (Nicolopoulos-Stournaras and Iles, 1983; Tosolini and Morris, 2012).

### Translational relevance

Neurological conditions that affect lower motor neurons are among the most debilitating motor disorders. Genetically based mouse models are currently available for conditions that directly affect the output of spinal cord motor neurons on the skeletal musculature. In particular, these include models of amyotrophic lateral sclerosis, i.e., the SOD-1-G93A and A315T-TDP-43 strains (Jackson Laboratory, Bar Harbor, ME, USA) (Kaspar et al., 2003; Ishiyama et al., 2004; Turner et al., 2009; Wegorzewska et al., 2009; Towne et al., 2010; Xu et al., 2010; Guo et al., 2011; Riboldi et al., 2011). Likewise, models of Duchenne’s muscular dystrophy, i.e., the various mdx strains (Jackson Laboratory, Bar Harbor, ME, USA) (Kimura et al., 2010; Pratt et al., 2013) and of SMA, i.e., the SMN^−/−^ strain (Hsieh-Li et al., 2000) are also available. Mice with these above-mentioned mutations display a typical motor phenotype with upper and lower limb deficits.

Several treatment strategies have been designed in an attempt to reverse the motor phenotype in these mutant mice. For instance, both pharmaceutical and cell-based therapies have been performed through different routes of administration (Raoul et al., 2005; Henriques et al., 2011; Teng et al., 2012). These therapeutic approaches have been shown to slow the progression of the motor phenotype, to increase lifespan of the affected animals but are yet to eradicate these conditions. Targeted delivery of therapeutic agents to motor neurons for the treatment of ALS-like phenotypes has also been achieved via intramuscular injections and the ensuing retrograde transport along the peripheral nerve (e.g., Kaspar et al., 2003; Wu et al., 2009; Calvo et al., 2011). Intramuscular injection and retrograde delivery of therapeutic molecules is a minimally invasive surgical procedure and, in combination with viral vectors, offers promising potential for translational gene therapy aiming at the restoration of motor function (Baumgartner and Shine, 1998; Giménez y Ribotta et al., 1998; Kaspar et al., 2003; Nakajima et al., 2008; Towne et al., 2010; Uchida et al., 2012; Benkhelifa-Ziyyat et al., 2013). The present anatomical investigation has clearly shown that targeting the full width of the muscles’ MEP region can maximize the success of somatic delivery of therapeutic molecules to spinal cord motor neurons. Thus, knowledge regarding the precise anatomical relationship between the different muscles of the mouse forelimb and the location of: (1) their MEP region and (2) the spinal cord motor neuron columns that supply them will prove to be valuable tools to further investigate new treatment for ALS and other related motor disorders.

## Conflict of Interest Statement

The authors declare that the research was conducted in the absence of any commercial or financial relationships that could be construed as a potential conflict of interest.
